# The Development of a Biomimetic Model of Bacteria Migration on Indwelling Urinary Catheter Surfaces

**DOI:** 10.3390/biomimetics9080491

**Published:** 2024-08-14

**Authors:** Yvonne J. Cortese, Joanne Fayne, Declan M. Colbert, Declan M. Devine, Andrew Fogarty

**Affiliations:** 1 PRISM Research Institute, Technological University of the Shannon: Midlands Midwest, N37 HD68 Athlone, Ireland; yvonne.cortese@tus.ie (Y.J.C.); declan.colbert@tus.ie (D.M.C.); 2 Teleflex Medical EMEA, N37 EC90 Athlone, Ireland; 3 Department of Bioveterinary and Microbial Sciences, Technological University of the Shannon: Midlands Midwest, N37 HD68 Athlone, Ireland; andy.fogarty@tus.ie

**Keywords:** biomimetic model, in vitro model, urethral catheterisation, bacterial migration, catheter-associated urinary tract infections

## Abstract

The aim of this study was to develop a novel biomimetic in vitro extraluminal migration model to observe the migration of bacteria along indwelling urinary catheters within the urethra and assess the efficacy of a prototype chlorhexidine diacetate (CHX) coating to prevent this migration. The in vitro urethra model utilised chromogenic agar. A catheter was inserted into each in vitro urethra. One side of the urethra was then inoculated with bacteria to replicate a contaminated urethral meatus. The models were then incubated for 30 days (d), with the migration distance recorded each day. Four indwelling catheter types were used to validate the in vitro urethra model and methodology. Using the biomimetic in vitro urethra model, *E. coli* and *S. aureus* migrated the entire length of a control catheter within 24–48 h (h). In the presence of a prototype CHX coating, full migration of the channel was prevented for 30 d. The results of this study support the hypothesis that catheter-associated urinary tract infections (CAUTIs) could be prevented by targeting catheter-mediated extraluminal microbial migration from outside of the urinary tract into the bladder.

## 1. Introduction

In a world where great leaps in medical science to fight a sudden pandemic are entirely possible, common healthcare-associated infections (HCAIs) continue to escape our grasp. Urinary catheters are ubiquitous in modern healthcare and catheter-associated urinary tract infections (CAUTIs) are one of the most common HCAIs [1]. CAUTIs directly impact patient morbidity and mortality rates, with indwelling urinary catheters (IDs) responsible for the majority of severe CAUTI complications including septicaemia, crystalline biofilm formation, bladder/kidney stones, pyelonephritis and septic shock [2].

### 1.1. CAUTI Initiation by Extraluminal Migration

IDs, when used long-term, provide a direct means of travel for microorganisms to enter the urinary tract from outside of the body. Microorganisms can move along the intra- and extraluminal surfaces of urinary catheters. Through capillary action and/or their own innate motility, microorganisms can ascend a catheterised urinary tract and manifest a CAUTI [3,4,5,6]. Infections initiated by bacterial migration on intraluminal surfaces typically originate from a contaminated urine collection system; however, with the advent of closed sterile urine collection systems in the 1950s, this occurs in the minority of cases [7,8]. In the majority of cases, extraluminal migration of bacteria is responsible for the ascension of bacteria from the urethral meatus along the periurethral mucous sheath to the bladder [7]. The urethral meatus plays host to its own diverse microbiota with a number of potentially pathogenic microbial species, both enteric and skin-borne species, that can cause CAUTI, including *Escherichia coli*, *Pseudomonas aeruginosa*, *Staphylococcal* sp., *Enterococcal* sp., *Proteus* sp., *Klebsiella* sp., *Citrobacter freundii*, *Providentia rettgeri* and *Candida* sp. [7,9]. The extraluminal surface of a catheter can become contaminated during insertion due to poor hand/meatal hygiene or due to the continued colonisation of the meatus from which pathogenic organisms can migrate [10,11,12].

### 1.2. CAUTI Prevention by Chlorhexidine

Numerous antimicrobials have been used to impregnate or coat catheters to prevent microbial colonisation or biofilm formation [1]. The majority of these solutions were focused on treating burgeoning infection. Other studies focused on preventing extraluminal migration, aimed at avoiding the initiation of an infection by blocking the entry of microbes into the urinary tract through either physical or chemical means. One antimicrobial compound of interest is chlorhexidine diacetate (CHX), a cationic bisbiguanide that acts through interaction between the cell membrane of microbes, which is negatively charged, and the positively charged functional group in the CHX structure, which ruptures the cell membrane of the microbe [13]. At low concentrations, CHX is bacteriostatic, with higher concentrations required to act as an irreversible bactericide [13,14]. Regarding urinary catheters, CHX has customarily been used as a cleanser for the meatal area during insertion and throughout the duration of a catheter’s placement or as an additive to urine-collecting systems [15,16]. In recent years, CHX has been trialled as a catheter additive or coating in the form of a varnish or encased in micelles, nanospheres and nanoparticles and was found to prevent bacterial proliferation for up to 28 days in some studies [17,18,19,20,21]. These studies argue that CHX could prove to be a useful antimicrobial in the fight against CAUTIs, and the prototype CHX coating used in this study aimed to provide protection against extraluminal migration for at least 30 days.

### 1.3. In Vitro Modelling of Extraluminal Migration

To assess extraluminal migration, one model has been described in past studies, i.e., the in vitro urinary tract model first described by Gaonkar et al. [22], later modified by Williams and Stickler [23] and Vargas-Cruz et al. [24]. This model utilised a short agar channel to qualify bacterial migration along an indwelling catheter. This model and subsequent adaptations inspired the modification of our previously published in vitro urethra model to visualise and quantify the extraluminal migration of bacteria on IDs [12]. This novel in vitro extraluminal migration model minimises contact between the agar and the enclosure, improving the directional control of bacterial migration. The model herein described contains a longer urethra channel (~140 mm compared to the 50 or 90 mm models described by [22,23,24]) to allow for quantification of migratory distances and improved visualisation of migratory patterns.

This article is based on Chapter 5 of the author’s PhD dissertation, which includes additional insights into the development and validation of the in vitro urethral extraluminal migration model and methodology [25].

## 2. Materials and Methods

### 2.1. Bacterial Strains, Media, Materials and Urinary Catheters

The bacterial strains used for antimicrobial and extraluminal migration testing were *E. coli* ATCC 25922 and *S. aureus* NCTC 12981. The chromogenic agars used for the in vitro extraluminal migration models were Harlequin™ *E. coli*/Coliform Agar (Neogen^®^, Lansing, MI, USA), which is selective for *E. coli* and other faecal coliforms, and CHROMagar™ Staph aureus agar (CHROMagar™, Paris, France), which is selective for *S. aureus* and other staphylococcal species. The prototype polymer coating, with or without CHX, was applied to each catheter’s extraluminal surface before sterilisation and subsequent testing. The formulation of said coating is protected intellectual property and, as such, its production and chemical constituents beyond the addition of CHX cannot be disclosed. The sample catheters used were all 14 French (4 mm Ø) and included uncoated silicone foley catheters (UNCs), silicone foley catheters fully coated in prototype coating with no CHX (−CHX), silicone foley catheters fully coated in prototype coating containing CHX (+CHX) and silicone foley catheters 50% coated in prototype coating containing CHX (+CHX50). UNCs acted as growth control to observe extraluminal migration in the absence of CHX or the polymer coating. The coating on each sample started ~10 mm from the meatal end of the model, allowing for a clear inoculation site free of coating. Sterile swabs used for in vitro model were paediatric swabs 2 mm in diameter.

### 2.2. In Vitro Extraluminal Migration Model

**Figure 1:**
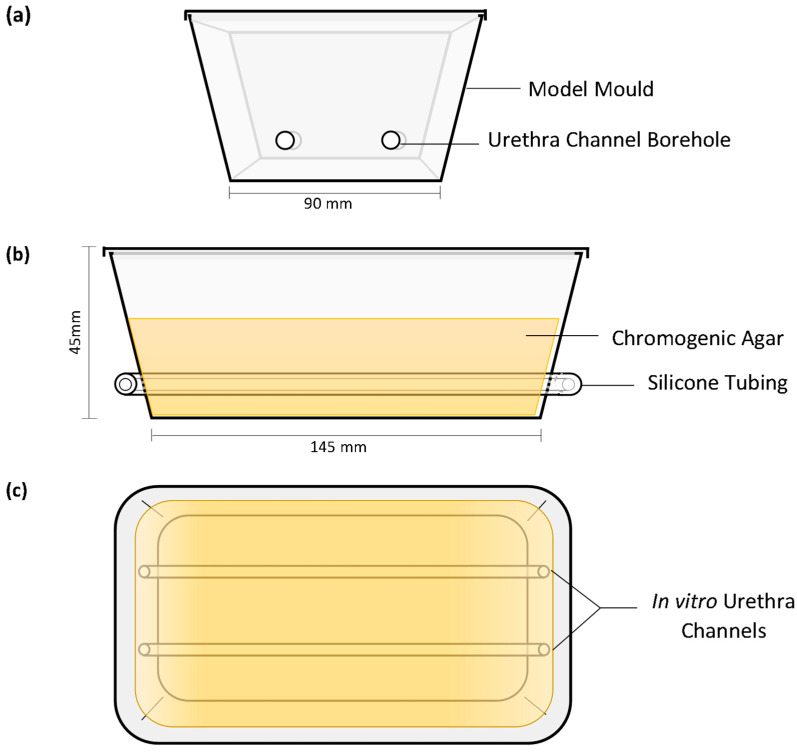

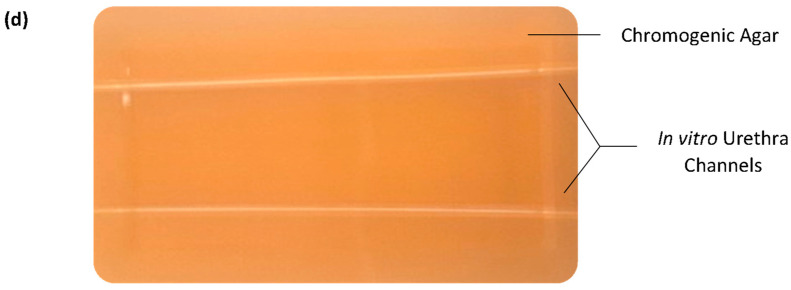
In vitro extraluminal migration model: end elevation (**a**), front elevation (**b**), planar view (**c**) and planar image (**d**).

The in vitro models were produced as previously described by Cortese et al. (2020) with minor changes to the model mould preparation [12]. The extraluminal migration model was thicker than the previously published model, utilising more agar to protect the model from excessive desiccation during the long incubation period. The bore holes of the mould were made on the short ends of the containers to allow for 140 mm length in vitro urethras; two channels were contained within each model (Figure 1). The in vitro urethra channels were 4 mm in diameter to provide an interference fit with catheters during testing. This ensured full contact between extraluminal surfaces and the in vitro urethral channel wall the entire length of the catheter. In vitro channels were spaced ~40 mm apart to prevent cross-contamination between channels as CHX migrated from the coating.

### 2.3. In Vitro Extraluminal Migration

**Figure 2:**
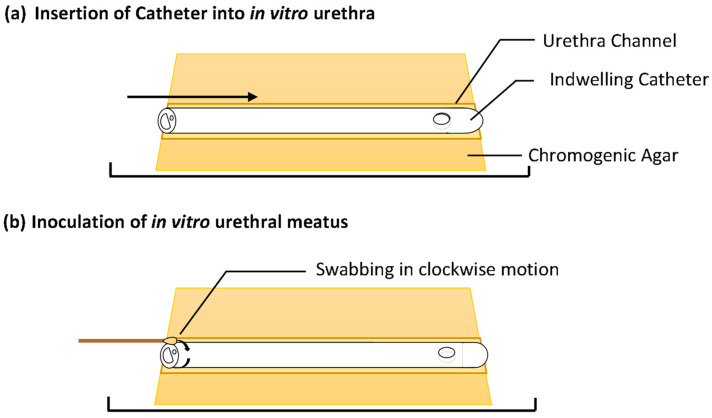

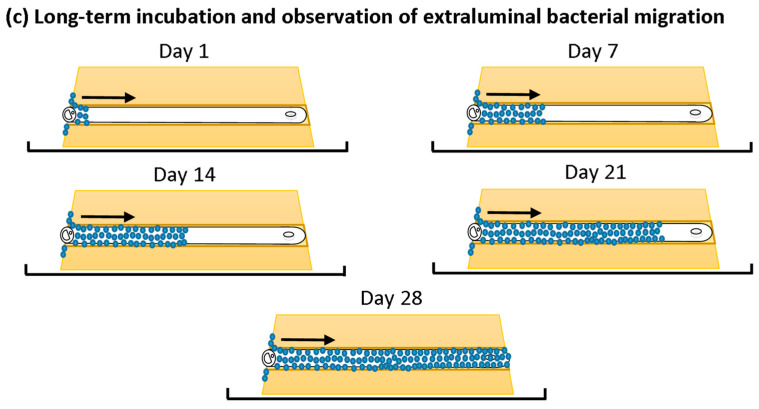
In vitro extraluminal migration model concept and methodology.

A single catheter was inserted into each in vitro urethra of the prepared models. A swab was then used to inoculate one side of each channel with a bacterial inoculum of either *E. coli* or *S. aureus* at a density of 10^5^–10^6^ Colony Forming Units (CFU) mL^−1^ to simulate a contaminated urethral meatus (Figure 2b). The models were then incubated for 30 d at 37 °C in a humidity-controlled environment. The progression of bacterial migration along the extraluminal surface was photographed and the migration distance was recorded on set days for 30 d (Figure 2c). This was repeated in six independent tests for each species. The normality of the data distribution was assessed with the Anderson–Darling test. As the data were not normally distributed, non-parametric testing was used. The reproducibility of the model and method was assessed by the non-parametric Kruskal–Wallis test to assess for variances between replicates. The non-parametric Mann–Whitney U test was used to compare test catheter samples vs. the growth control and determine if there was any significant difference in extraluminal migration.

### 2.4. Serial Plate Transfer Test (SPTT)

**Figure 3:**
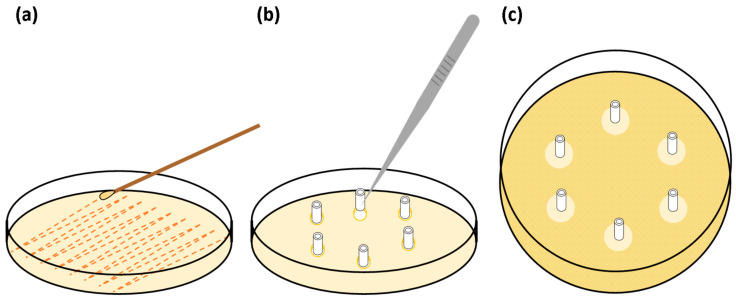
Illustration of the serial plate transfer test (SPTT) method: (**a**) inoculation of entire agar surface, (**b**) placing of catheter samples into wells in agar surface, (**c**) zones of inhibition around samples after incubation.

Based on a modified method described by Fisher et al. (2015), three sets of sextuplet, 10 mm long segments of UNCs and −CHX and +CHX catheters were prepared [26]. A bacterial inoculate at a density of ~1.5 × 10^8^ CFU mL^−1^ or a 0.5 McFarland of either *E. coli* or *S. aureus* was also prepared in phosphate-buffered saline by the McFarland method. A sterile swab was used to inoculate the entire surface of Mueller Hinton agar plates. Six 4 mm Ø wells were created in the agar surface of each plate and samples were aseptically placed in the wells. The plates were not inverted and were incubated at 37 °C for 24 h, after which the zone of inhibition around each sample was recorded in millimetres. This process was repeated each day for 30 d, transferring the same samples to new wells in freshly inoculated plates each day (Figure 3).

### 2.5. Minimum Inhibitory Concentration (MIC)

Two bacterial inocula ~1.5 × 10^8^ CFU mL^−1^ of *E. coli* and *S. aureus* were prepared in Mueller Hinton Broth (MHB). This was serially diluted to a final in-well bacterial density of 5 × 10^5^ CFU mL^−1^. A 0.8 µg mL^−1^ CHX stock solution was prepared in sterile deionised water (dH_2_O) and later diluted in MHB in two-fold dilutions to form an in-well range of 0.4 µg mL^−1^ to 0.0007 µg mL^−1^. In a 96-well plate, one column acted as a sterility control with 100 µL of sterile MHB added to each well; for the growth control, 50 µL of MHB and 50 µL of inoculum were added. To each test well, 50 µL of inoculum was added and then 50 µL of the corresponding concentration of CHX was added. This was repeated in quadruplicate wells for each strain and repeated in two independent tests. The plates were then sealed in Parafilm to prevent evaporation and incubated at 37 °C for 18 h in an oscillating incubator. After incubation, 10 µL of 0.02% resazurin was added to each well and incubated with oscillation for 2 h at 37 °C. The fluorescence of each well was then quantified by a microplate reader set for excitation at 530 nm and emission at 590 nm to detect the metabolically reduced fluorescent resorufin. All wells containing chlorhexidine diacetate were compared to the growth controls to calculate the reduction in cellular viability.

### 2.6. Drug Release Trial

The peak absorbance (λ_max_) of CHX was first determined by a wavelength scan of a stock solution at 2 ng mL^−1^ CHX dissolved in dH_2_O. The λ_max_ wavelength was then used for all further readings. A set of CHX standards were then prepared with a range of 2 ng mL^−1^ to 0.22 ng mL^−1^. The absorbance of each standard was then read at 230 nm. A standard curve was plotted and used to calculate the drug concentration from the absorbance values. To assess the release rate of CHX from the polymer coating, 10 mm triplicate samples of UNCs and −CHX and +CHX catheters were prepared. Each sample was placed in 10 mL of dH_2_O in a T25 culture flask. The flasks were incubated in darkness at 37 °C for 30 d in an oscillating incubator. Each day, 1 mL was removed from each flask to read the drug concentration in the dH_2_O by UV absorption; 1 mL of fresh dH_2_O was then added to each flask before being replaced in the incubator. The concentration of the drug on each day versus the cumulative release was then plotted.

## 3. Results

**Figure 4:**
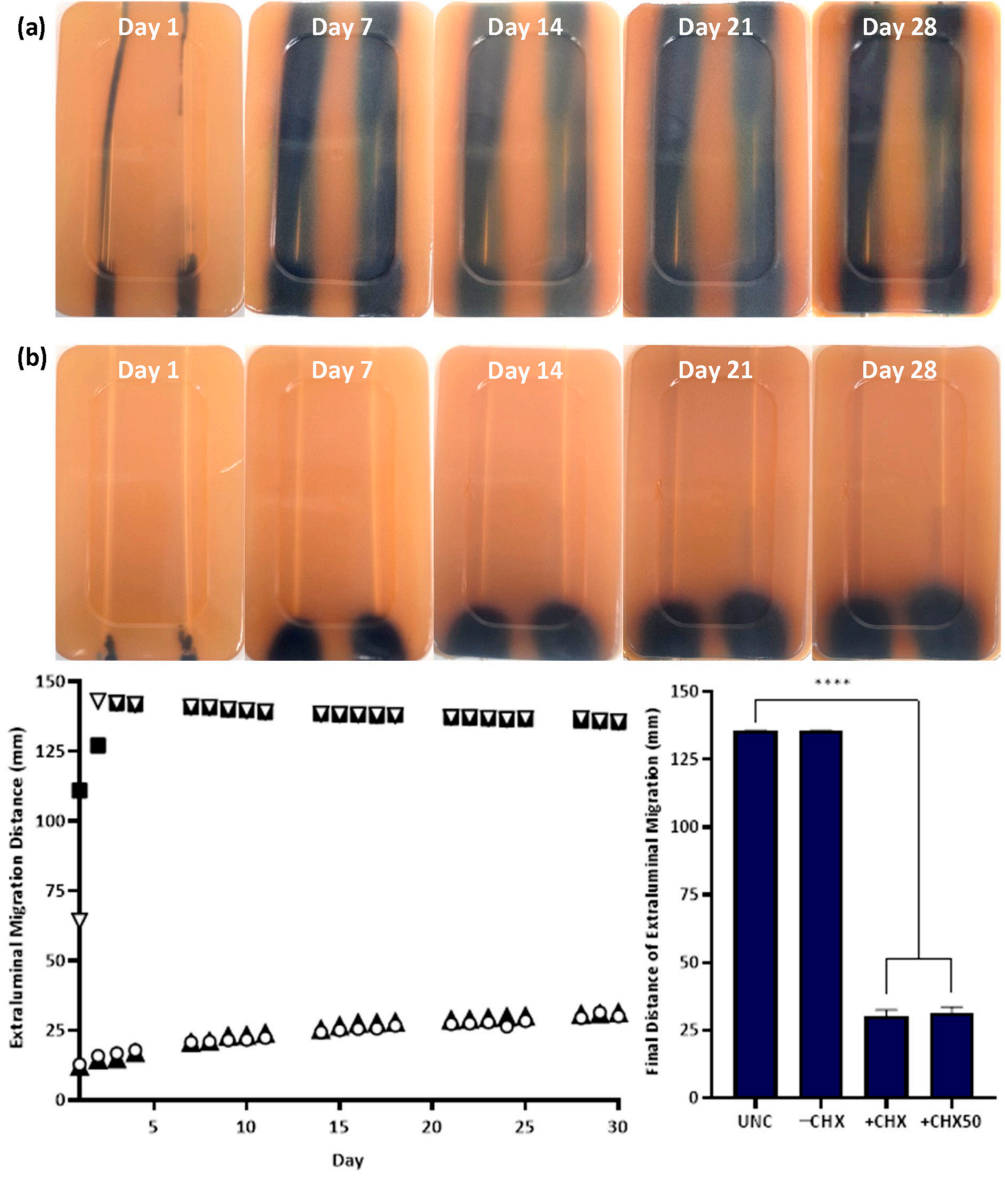
Extraluminal migration of *E. coli* ATCC 25922 on UNCs (**a**, left, ▽) and −CHX (**a**, right, ■), +CHX (**b**, left, ○) and +CHX50 (**b**, right, ▲) catheters over 30 days. Bacterial growth is *E. coli* grown in Harlequin™ *E. coli*/Coliform Agar (**a**,**b**). Error bars represent standard error of the mean (n = 6). **** *p* ≤ 0.0001.

**Figure 5:**
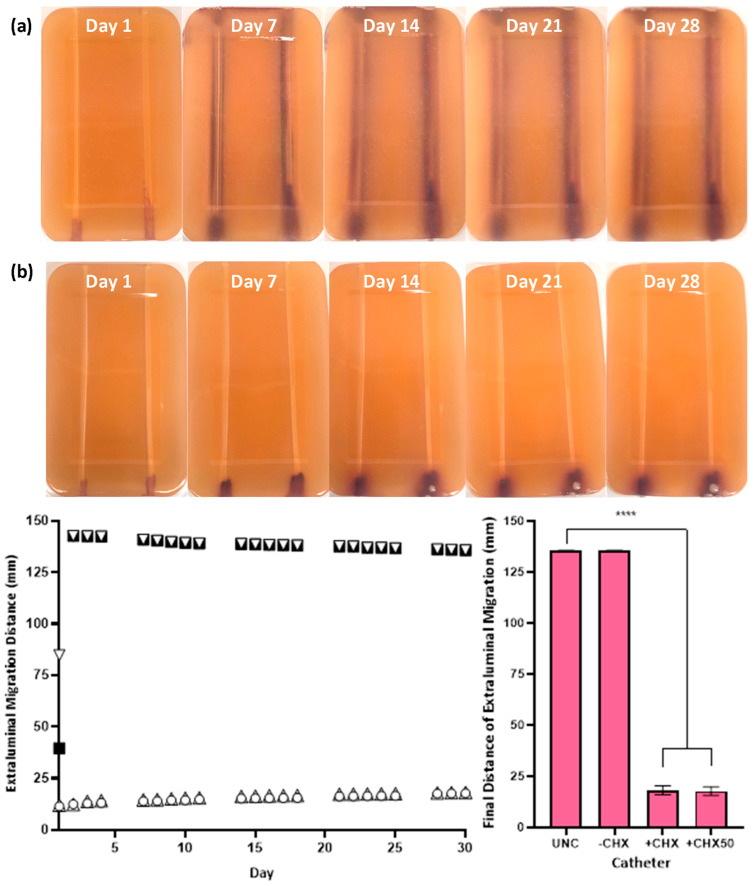
Extraluminal migration of *S. aureus* NCTC 12981 on UNC (**a**, left, ▽) and −CHX (**a**, right, ■), +CHX (**b**, left, ○) and +CHX50 (**b**, right, ▲) catheters over 30 days. Bacterial growth is *S. aureus* grown in CHROMagar™ Staph aureus agar (**a**,**b**). Error bars represent standard error of the mean (n = 6). **** *p* ≤ 0.0001.

The in vitro extraluminal migration model was validated through twelve independent tests (six with either species). No significant difference was observed in the control and CHX-free samples tested (UNC and −CHX) between replicates (*p* ≥ 0.8510), demonstrating the reproducibility of the model and test methodology. Complete migration from the meatal side of the model to the bladder side occurred within 24–48 h in the absence of CHX for UNCs and −CHX catheters with either bacterial species (Figure 4 and Figure 5). With the inclusion of CHX, +CHX and +CHX50 catheters prevented extraluminal migration for 30 d, demonstrating a significant difference from the non-antimicrobial samples (*p* < 0.0001). No significant difference was observed in migration prevention efficacy between +CHX and +CHX50 catheters with either bacterial species (*p* = 0.1084).

**Figure 6:**
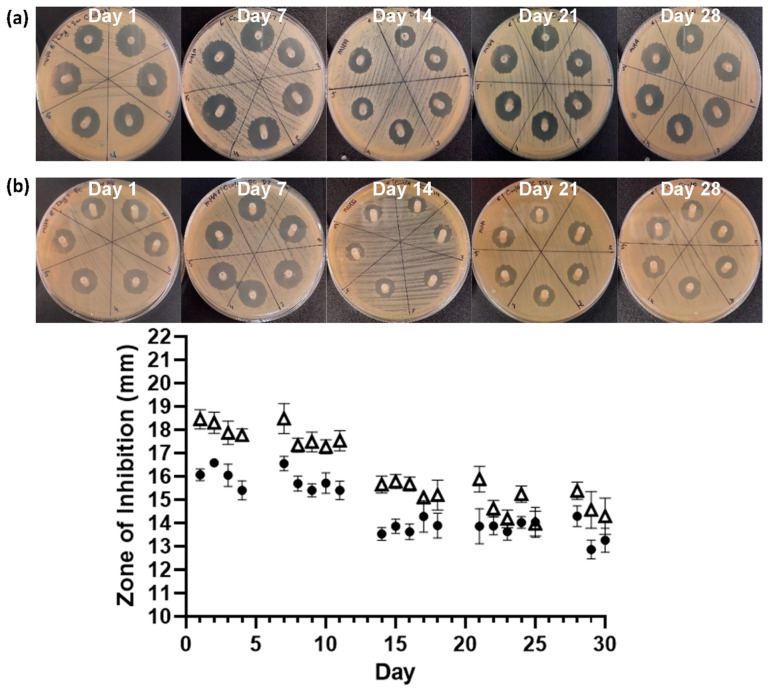
Images of the serial plate transfer test for +CHX catheter when exposed to *S. aureus* NCTC 12981 (**a**, △) and *E. coli* ATCC 25922 (**b**, ●). Error bars represent standard deviation, n = 6.

**Figure 7:**
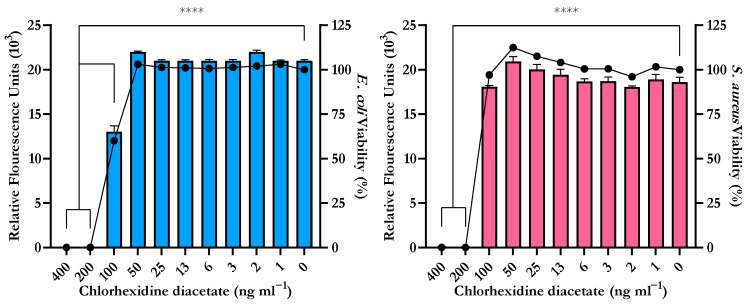
Minimum Inhibitory Concentration (MIC) of chlorhexidine diacetate for *E. coli* ATCC 25922 (**left**) and *S. aureus* NCTC 12981 (**right**) determined by the reduction of resazurin to resorufin correlated to reduction in cellular viability (●). Error bars represent standard deviation, n = 8, **** *p* ≤ 0.0001.

**Figure 8:**
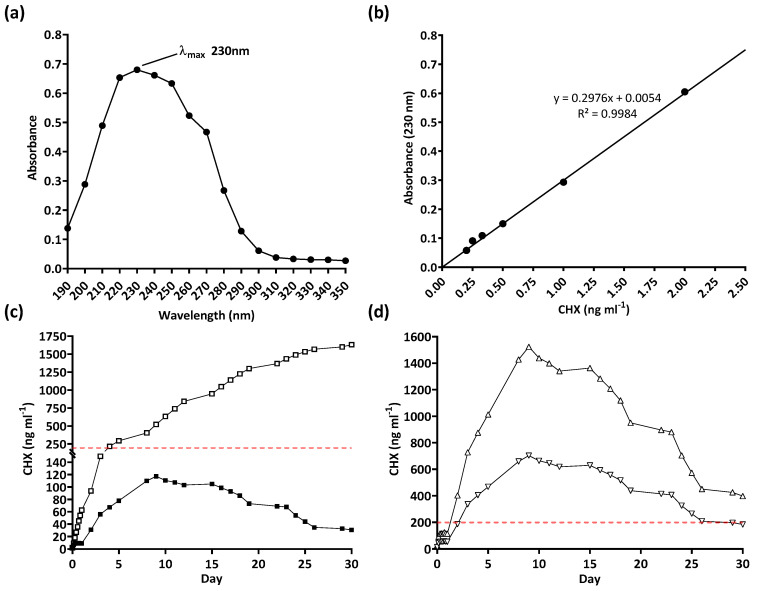
Drug release trial of chlorhexidine diacetate (CHX): (**a**) determination of λmax, (**b**) standard curve, (**c**) 30-day real-time (■) and cumulative (☐) release of CHX from coated 10 mm samples, and (**d**) predicted 30-day CHX release from fully coated catheters (+CHX, △) and 50% coated catheters (+CHX50, ▽). Dashed line indicates the Minimum Inhibitory Concentration of CHX.

The serial plate transfer test demonstrated consistent inhibition of bacterial growth by CHX-containing samples (+CHX) for at least 30 days and no significant difference (*p* ≥ 0.1170) was observed between replicates with either species (Figure 6). The MIC of CHX was determined to be 0.2 µg mL^−1^ for both *E. coli* and *S. aureus* through the microtiter MIC assay (Figure 7). The drug release trial confirmed the extended release of CHX from the prototype coating of +CHX catheters for ≥30 d, with CHX concentrations peaking on day 9 at 0.12 µg mL^−1^ (Figure 8).

## 4. Discussion

The primary aim of this study was to develop an in vitro model and methodology focused on the visualisation and quantification of extraluminal bacterial migration on urinary catheters as a target for CAUTI prevention. In our previous study, we focused on the movement of bacteria from the meatus to the bladder by friction-mediated pathogen displacement during catheter insertion. We demonstrated that meatal contamination during catheter placement can directly contribute to CAUTI manifestation, and that reducing/eliminating friction could be a target to prevent CAUTIs [12]. With this in mind, the in vitro extraluminal migration model was designed to further study meatal colonisation as an origin for CAUTI and the role that catheters play in promoting the movement of bacteria into the urinary tract.

The in vitro extraluminal migration model described in this study was validated through 12 independent tests with two bacterial strains to ensure the reproducibility of the model and associated methodologies as well as the versatility with both Gram-negative and -positive species. No significant difference was detected between replicate growth controls or non-antimicrobial samples (UNC and −CHX catheters) with either species tested (*p* ≥ 0.8510). While there was a statistically significant difference detected between the replicates of +CHX and +CHX50 catheters, there was no noticeable difference in overall growth patterns. The statistically significant differences observed between replicate +CHX and +CHX50 catheters is most likely due to biological variance in response to CHX exposure. *E. coli* and *S. aureus* were chosen to validate the model as they are standard species for studying antimicrobial susceptibility; they are representative of clinically relevant CAUTI strains; they are representatives of Gram-negative and -positive species; and they exhibit active or passive motility, respectively [27,28,29]. It should be noted that *E. coli* ATCC 25922 and *S. aureus* NCTC 12981 are both types of strains and not CAUTI clinical isolates; as such, the results of this study may not be representative of the characteristics of uropathogenic strains. The relevance and translational potential of the model could be increased by the inclusion of relevant uropathogenic strains, e.g., *P. mirabilis*, *C. albicans*, *P. aeruginosa*, etc. However, this was beyond the scope of this study.

Utilising the in vitro extraluminal migration model, extraluminal migration, in the absence of CHX, from the meatal side of the model to the bladder was observed to occur within 24–48 h when using either *E. coli* or *S. aureus* (UNCs and −CHX catheters). While the speed at which *E. coli*, an actively motile species, traversed the catheter within the in vitro urethra was unsurprising, *S. aureus*, a passively motile species, was also consistently observed to migrate the full distance within the same time period [4]. Traditionally, *S. aureus* has been classified as a passively motile species, with movement associated with sliding motility. Recent studies have observed some strains of *S. aureus*, in specific environments, engaging in active gliding motility with organised directional movement from a central colony [29,30]. While this form of active motility could be responsible for the relatively quick ascension of *S. aureus* in the in vitro urethra, another factor to consider is the aid of capillary action, which could have exacerbated passive sliding motility. It should be noted that the in vitro urethra extraluminal migration model is not an anatomically correct model as there in no flow of moisture within the in vitro urethra channel. Small amounts of urine can escape through the urethra when a catheter is in place, and this in conjunction with urethral mucosa secretions creates a moist environment between the catheter and urethral walls which could facilitate changes in bacterial migration patterns and speeds; so, further in vivo testing is needed to draw any firm conclusions [31].

In the presence of the novel CHX coating, complete extraluminal migration to the bladder side was prevented for 30 days with either species. There was a significant reduction in the final bacterial migration distance on both +CHX and +CHX50 catheters when compared to either UNCs or −CHX catheters (*p* ≤ 0.0001). In the presence of CHX, on either +CHX or +CHX50 catheters, *E. coli* migrated an average of 30.72 ± 11.72 mm over the 30 d period and *S. aureus* reached an average of 17.94 ± 4.23 mm in the same time span. This result gives an estimated migration speed of ~1 mm d^−1^ for *E. coli* and ~0.6 mm d^−1^ for *S. aureus*. There were no significant differences in final migration distances between +CHX or +CHX50 catheters, indicating that fully coating a catheter in the CHX coating is not necessary to achieve migration prevention for 30 d (*p* ≥ 0.1084). Reducing the amount of coating to 50% (+CHX50) could allow for an overall cheaper product for patients and a reduction in CHX exposure without compromising on infection protection.

The long-term release of CHX from the novel coating is supported by the SPTT results, which demonstrated that the antimicrobial-coated catheter segments exhibited antimicrobial activity for at least 30 d. In the SPTT, *S. aureus* was observed to have a greater sensitivity to CHX, which may be responsible for the ~13 mm lower average final migration distance of *S. aureus* in the in vitro extraluminal migration model (Figure 6).

The drug release trial also supports the extended release of chlorhexidine for at least 30 d. It should be noted that the drug release trial was limited to 10 mm segments of coating. During the 30 days, the level of chlorhexidine released, on any given day, did not reach or exceed the MIC of both bacterial species tested (0.2 µg mL^−1^). However, as there was no significant difference between replicates observed (*p* = 0.8092), the results obtained could be used to predict the release of chlorhexidine when larger sections of the catheter are coated. To that end, both +CHX and +CHX50 catheters were predicted to reach the MIC within 24–48 h if the experiments were to be repeated (Figure 8d).

In consideration of the results of this study, it can be concluded, within an in vitro setting, that the novel CHX-containing polymer coating can prevent extraluminal bacterial migration, of the strains studied, on urinary catheters for ≥30 d. While these results are promising, there are two major factors that need to be considered in the future development of CHX as a catheter coating. First is the potential inactivation of CHX by anionic compounds originating in the catheter materials, excretions of the urethral mucosa, or from lubricants/disinfectants used during the insertion process. The cationic properties of CHX can lead to inactivation or precipitation in the presence of anionic substances that could ultimately reduce its antimicrobial efficacy [32]. More research is needed to elucidate the effects, if any, of urethral mucosa secretions on the potential inactivation of CHX. The second consideration needed for CHX as a urinary catheter coating is the safety of long-term in situ use. CHX, at therapeutic levels, is considered safe for use in cosmetics, medicines and medical devices with few toxicological concerns and low levels of mammalian toxicity [33,34]. In consideration of patient safety, the main concern with CHX use is the potential for sensitisation and anaphylactic allergy development [34]. With these considerations in mind, CHX as a catheter coating warrants further study; however, due to its potential for inducing allergic responses in patients and its sensitivity to anionic compounds, its use in clinical settings should be monitored to avoid allergic sensitisation and inactivation.

The result of this study emphasises the need for further investigation into the mechanisms involved in the initiation of CAUTIs. Instead of treating an infection or minimising its impact, the goal should be preventing the infection taking hold in the first place. The observed speed at which both actively and passively motile species can ascend the urethra and gain access to the bladder is concerning. These findings could imply that any contamination of the meatal area could lead to the initiation of an infection. Regardless of patient hygiene, it is not possible to fully eradicate pathogenic meatal microflora. Potentially the best strategy towards preventing infection is inhibiting their ability to use an ID as a bridge into the urinary tract. With the use of a CHX coating, the extraluminal migration of meatal bacteria was prevented for up to 30 days. If effective in clinical settings, this could greatly increase the amount of time a patient can be catheterised before the onset of bacteriuria or infection. Targeting the prevention rather than the treatment of infection could also have positive effects towards reducing the number and amount of antibiotics needed for patient care. Antibiotic-resistant CAUTI strains are on the rise in clinical settings, so any strategy that minimises antibiotic use is preferable [35].

As previously stated, further research is needed into extraluminal migration to determine if this preventative strategy could lower or eliminate the risk of CAUTIs. While the in vitro extraluminal migration model provides a robust tool to screen novel antimicrobial IDs, it does present limitations when considering its anatomical and clinical relevancy. To expand this study further in the future, at an in vitro level, it will be important to include clinically relevant uropathogenic strains. In particular, it would be of great interest to study the extraluminal migration abilities of *P. miralibis* as its unique raft forming swarming behaviour may prove more challenging to control [36]. Additionally, expanding this study to include an array of different antimicrobial agents including not just bactericidal compounds but also antifouling compounds may provide more information on the impact different antimicrobial strategies have on extraluminal migration. Finally, to increase the translational potential of this study, moving to an in vivo model should be carried out to prove that the findings of this study translate to a clinical environment.

If early extraluminal migration can be delayed or prevented, the length of time a patient can be safely catheterised before infection occurs could be improved or, in a best-case scenario, the initiation of the infection can be avoided entirely, improving clinical outcomes for patients. We envisage that the in vitro urethra extraluminal migration model will provide a robust tool to further study bacterial migration on urinary catheters and other medical devices as a target to prevent HAIs.

## Data Availability

The data that support the findings of this study are available from the corresponding author, Dr Declan Devine, upon reasonable request.
